# Infant dietary patterns and early childhood weight outcomes: a secondary analysis from the Starting Early Program Trial

**DOI:** 10.21203/rs.3.rs-8077856/v1

**Published:** 2025-11-25

**Authors:** Lauren Berube, Christina Kim, Andrea Deierlein, Kathlee Woolf, Mary Jo Messito, Rachel Gross

**Affiliations:** New York University School of Global Public Health; NYU Grossman School of Medicine; New York University Grossman School of Medicine; New York University Grossman School of Medicine

**Keywords:** childhood obesity, dietary patterns, weight-for-age, infant feeding, prevention

## Abstract

**Background:**

Limited studies assess how infant dietary patterns impact child weight. The Starting Early Program (StEP) promotes healthy nutrition during pregnancy and infancy and leads to healthier child weight, but whether dietary patterns contribute to weight or mediate StEP weight outcomes has not been studied.

**Objectives:**

This secondary analysis identified infant dietary patterns in StEP, determined associations between dietary patterns and child weight outcomes, and examined whether dietary patterns mediated the relationship between StEP and child weight.

**Methods:**

Data were from 377 mother-infant dyads enrolled in a randomized trial testing the efficacy of StEP. Infant diet was assessed at 10 months using an interviewer-administered 24-hour recall, and dietary patterns were identified using latent class analysis. Child weights were abstracted from medical records at 12, 24, and 36 months. Associations between infant dietary patterns and weight-for-age z-score (WFAz) and likelihood of being classified as overweight (WFA ≥85^th^ percentile) were assessed using multivariable linear and logistic regression models. Mediation analyses were used to assess intervention effects on WFAz via impacts on infant dietary patterns.

**Results:**

Four classes of infant dietary patterns were identified, characterized by differences in milk feeding and complementary food type and variety: Breastfed-High variety; Formula fed-High variety; Formula fed-Low variety; and Mixed fed-Low variety. Compared to the Breastfed-High variety class, infants in the Formula fed-Low variety class had higher WFAz and were more likely to be classified as overweight at 24 and 36 months. Participation in StEP increased membership in Breastfed-High variety, which mediated the association between StEP and lower WFAz at 24 months.

**Conclusions:**

Infant dietary patterns were identified and associated with child obesity risk. StEP promoted an infant dietary pattern that was most consistent with infant feeding guidelines, which mediated intervention effects on child weight.

## Introduction

Nearly 13% of children aged 2 to 5 years in the United States (U.S.) are classified as having obesity, and 16% aged 2 to 19 years are classified as overweight (1, 2). Childhood obesity persists throughout the lifespan and increases risks of comorbidities like type 2 diabetes and cardiovascular disease,(3) highlighting the need for early prevention. The time from conception until age 2 years is considered a critical period for the development of obesity when nutrition, lifestyle and environmental exposures influence later behaviors and health outcomes (4). Dietary intake during this period contributes to childhood obesity risk (5, 6). Healthy infant feeding practices not only support optimal growth and development (7), but also establish flavor preferences and eating behaviors that are often maintained throughout childhood (8), making infancy an important period to promote healthy dietary behaviors.

Dietary recommendations for infants promote breastfeeding or iron-fortified formula feeding through the first year of life and the introduction of a variety of nutrient-dense complementary foods (e.g., fruits and vegetables) around 6 months when developmentally ready, with avoidance of cow’s milk, juice, sugar-sweetened beverages, and foods with added sugars, saturated fat, and sodium (9, 10). However, data from national surveys indicate that many infants and toddlers in the U.S. are not fed according to these recommendations. Approximately 40% of infants and toddlers do not meet recommended intakes for fruit, and nearly 90% do not meet recommendations for total vegetables (9). Additionally, 17% of infants consume cow’s milk and 27%, 34%, and 77% consume juice, sweets/sugar-sweetened beverages, and savory snacks, respectively (11, 12). These high rates of suboptimal feeding practices highlight the need to understand how early life dietary patterns are related to child weight outcomes, particularly in populations that are disproportionately affected by childhood obesity.

Considerable research has examined how individual infant feeding practices, including exclusivity, duration, and intensity of milk-based feedings (13, 14) and adherence to complementary food recommendations (15, 16), contribute to child weight outcomes. However, studying individual aspects of infant feeding does not sufficiently account for the whole diet and the complex interactions of the multiple dietary components consumed (17). Infant dietary patterns measure the totality of the diet, considering milk feeding type and amount and variety of complementary foods, but limited studies have assessed how infant dietary patterns impact later child weight status. Latent class analysis (LCA), a person-centered data reduction approach, has been used to identify infant dietary patterns among primarily non-Hispanic white middle- or higher-income populations (18–20). Patterns characterized by foods high in energy density or low in variety were associated with greater weight at 12 months (18), 24 months (20), and 6 years (19). Children from Hispanic and lower-income populations experience the highest rates of obesity (1), yet there is a lack of research investigating associations between infant dietary patterns and weight in these populations.

The Starting Early Program (StEP) is a child obesity prevention intervention that supports optimal child feeding and lifestyle behaviors for Hispanic and low-income families. StEP reduces child weight trajectories through 24 months (21) and improves maternal infant feeding knowledge, styles, and practices (including increasing breastfeeding and decreasing juice) (22). Despite these positive impacts on feeding behaviors, it is unclear how StEP impacts infant *dietary patterns* and whether these dietary patterns contribute to later child weight outcomes. To address these gaps, the current study used LCA to identify distinct classes of infant dietary patterns in the StEP cohort. We aimed to 1) examine associations between dietary patterns and child weight and 2) examine potential impacts of the StEP intervention on dietary patterns and whether these patterns mediate the relationship between StEP and child weight.

## Methods

### Study design

Data for this secondary analysis were from the StEP trial, a randomized controlled trial to test the impact of a primary care-based child obesity prevention intervention beginning during pregnancy on early child weight outcomes in Hispanic families with low-incomes (ClinicalTrials.gov Identifier: NCT01541761) (23). Ethics approval was obtained from the Institutional Review Boards of New York University Grossman School of Medicine (Institutional Review Board number: 10–02175) and the New York City Health + Hospitals (System to Track and Approve Research number: StudyTX00001047), and trained bilingual research assistants obtained written informed consent from all participants. A detailed study design of StEP has been published elsewhere (21, 24).

### Study sample

At 28 to 32 weeks gestation (baseline), participants were recruited from prenatal clinics affiliated with a large hospital system in New York City. Patients with self-reported Hispanic/Latina ethnicity, fluent in English or Spanish, ≥ 18 years of age with a singleton uncomplicated pregnancy, and planned continuation of pediatric care at the study site were eligible. Participants with a history of severe medical or psychiatric illness or drug/alcohol use disorder, as well as those with fetal abnormalities detected via ultrasound, were excluded.

For this secondary analysis, participants with a dietary recall at the 10-month assessment were included in the LCA. Infants ≥ 12 months at the assessment were excluded, as dietary recommendations change at 12 months (9). Children with ≥ 1 recorded weight at 12, 24, or 36 months were included in the analysis of dietary patterns and child weight.

### Starting Early Program intervention

Participants were randomly assigned to either the standard care or the StEP group. Standard care received standard prenatal and pediatric primary care. StEP received standard primary care visits plus individual counseling sessions after 32 weeks gestational age and at infant age 2 to 3 days, and 15 group nutrition and parenting classes from infant age 1 to 33 months delivered by registered dietitians and certified lactation counselors. Individual counseling sessions reviewed breastfeeding support, and group session topics included feeding, activity, and parenting.

### Assessments

#### Infant dietary assessment

##### 24-hour dietary recall.

At infant age 10 months, participants completed a 24-hour dietary recall modified to collect information about infant feeding, including detailed information on the type (breastmilk vs formula) and of mode (breast vs bottle) of milk feeding, consumption of complementary foods, and self-feeding. Mothers recalled what their infants consumed during the previous day, or, if the previous day was not reflective of usual intake, for a typical 24-hour period, providing the day of the week that was being recalled. Trained bilingual research assistants asked the time and mode (breast or bottle) of feeding, and whether the infant was given food or other beverages. For bottle feeds, mothers reported the milk type (expressed breastmilk, formula, or something else), whether anything was added, and, if so, what was added. For food or other beverages, mothers described the type and quantity of each food item given, how it was prepared, how it was fed, and how much the infant ate. These prompts were repeated to capture all feedings in one 24-hour period and recorded in a paper-based format, which was entered into the Automated Self-Administered 24-Hour Dietary Assessment 2014 (ASA24) tool, developed by the National Cancer Institute (NCI, Bethesda, MD) (25). Food Pattern Equivalents Database (FPED) values were generated using a SAS program available from the NCI to estimate intake of food groups (26).

##### Dietary intake variables.

Dietary variables were created following guidelines from the Child and Adult Care Food Program (CACFP) Infant Meal Patterns for 6- to 11-month infants (27) and informed by prior methods (18, 20). Using the FPED values, dietary variables were dichotomized to identify daily intake of breastmilk; formula; cow’s milk; other dairy (e.g., yogurt, cheese, cottage cheese); infant cereals; grains (e.g., rice, pasta, bread, crackers, Cheerios); total vegetables (non-starchy and starchy); whole fruits; fruit juice; animal proteins (e.g., meat, poultry, fish, whole egg); legumes (e.g., cooked dry beans, cooked dry peas); and empty calorie foods (e.g., pastries, sugary cereals, salty snacks, French fries). Legumes were separated from animal proteins due to the more frequent consumption of legumes in diets of infants from Hispanic families (28). Since the recall did not differentiate between 100% fruit juice and other juice drinks, any juice consumed was included in the juice category.

##### Breastfeeding or formula feeding intensity.

To determine predominant feeding mode, breastfeeding and formula feeding intensity were estimated as [breastmilk feeds OR formula feeds/(breastmilk feeds + formula feeds + cow’s milk feeds)*100] (29). Infants with breastfeeding or formula feeding intensity ≥ 80% were considered predominantly breastfed or formula fed, respectively. Infants who were neither predominantly breastfed nor formula fed were considered mixed fed (e.g., 50% of feedings from breastmilk, 50% from formula).

##### Additional dietary variables.

Cow’s milk, other dairy, infant cereal, grains, fruits, vegetables, animal proteins, legumes, juice, and empty calorie foods were dichotomized based on the infant meeting or not meeting the recommended number of daily servings (27). For cow’s milk, juice, and empty calorie foods, the recommendation is 0 servings. For infant cereal, other dairy, grains, animal proteins, and legumes, the recommendation is ≥ 1 serving (> 0 tablespoons per serving). For fruits and vegetables, the recommendation is ≥ 4 servings total (> 0 tablespoons per serving). Separate categories were created for fruits and vegetables, and the recommendation was split into ≥ 2 servings of fruits and ≥ 2 servings of vegetables.

#### Child Weight Outcomes

Child weights were obtained from the electronic medical record (EMR) of primary care well-child visits at 12, 24, and 36 months. Sex-specific weight-for-age z-scores (WFAz) at each timepoint was calculated using the World Health Organization Anthro macro (30). WFA ≥ 85th percentile was defined as overweight. We used WFA instead of weight-for-length due to identified inaccuracies in EMR-obtained length and height measurements, detailed elsewhere (21).

#### Covariates

Maternal age, parity, country of birth, and education were collected from the baseline assessment. Marital status, employment, and participation in the Special Supplemental Nutrition Program for Women, Infants, and Children (WIC) were completed at the 10-month assessment. The U.S. Adult Food Security Survey Module assessed food security at 10 months; participants who reported ≥ 3 food insecure conditions were categorized as experiencing household food insecurity (31). Maternal anthropometric measurements were from the EMR. Pre-pregnancy body mass index (BMI, kg/m^2^) was calculated using measured weight (≤ 13 weeks gestation) and height or, if unavailable, self-reported values from the baseline assessment. Gestational weight gain (kg) was calculated by subtracting pre-pregnancy weight from delivery weight. Delivery mode, infant sex, and birthweight were from the EMR. Age at introduction of complementary foods and childcare was reported at 10 months.

#### Statistical Analysis

To identify discrete, mutually exclusive latent classes of infant dietary patterns, LCA was performed using Mplus version 8.3 (Muthén & Muthén, Los Angeles, CA). We selected LCA rather than an index-based approach because the StEP curriculum did not instruct participants to follow a single dietary pattern; rather, StEP targeted key obesity-related, age-appropriate feeding practices, including milk feeding type (promoting breastfeeding or counseling on healthy formula feeding) and complementary feeding (encouraging a variety of nutrient-dense foods rather than empty calorie foods). Additionally, families who participated in StEP were culturally diverse, and LCA helps identify differences in food consumption across diverse cultural groups. Latent class models with two through five latent classes were compared. The best fitting model was selected based on the Akaike’s Information Criterion (AIC), the Bayesian Information Criterion (BIC), the adjusted Bayesian Information Criterion (ABIC), a parametric bootstrapped likelihood ratio test (BLRT), relative entropy, the number of individuals within each class, and latent class interpretability (32). After identifying the optimal number of classes, participants were categorized into the class that corresponded to their highest-class membership probability.

Descriptive analyses, regression models, and mediation analyses were performed using the Stata Data Analysis and Statistical Software Version 14.2 (StataCorp LLC, College Station, TX). Maternal and infant characteristics were described by latent class membership using one-way analysis of variance or chi-squared tests. Linear regression models were used to analyze the association between latent class membership and WFAz at 12, 24, and 36 months, and logistic regression models were used to analyze the association between latent class membership and likelihood of being classified as overweight at the same timepoints. Regression analyses were adjusted for infant sex, birthweight, delivery mode, maternal age, marital status, education, pre-pregnancy BMI, gestational weight gain, parity, country of birth, household food insecurity, WIC participation, age of introduction of complementary foods, and study group assignment. We ran mediation analyses to determine whether the StEP intervention effects on child WFAz were mediated through 10-month dietary patterns. Since infant dietary patterns were measured at 10 months and milk feeding remains a significant source of energy for infants through 12 months, we expected that any intervention impacts on infant diet would likely mediate intervention effects on weight beyond 12 months. Therefore, the 24-month mediation analysis was adjusted for 12-month WFAz. We did not test for mediation at 36 months because StEP only impacted WFAz through 24 months.

## Results

**Figure 1:**
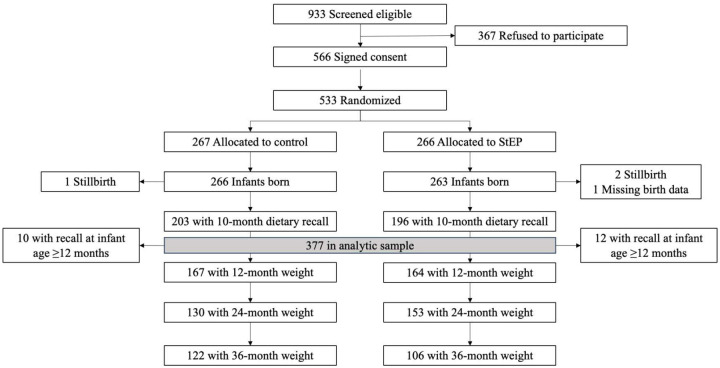
Participant flowchart. StEP: Starting Early Program.

Of 933 eligible mothers, 566 signed consent, and 533 were randomized. Of those, 399 completed recalls at 10 months. We excluded 22 infants (5.5%) for being ≥ 12 months at the time of assessment. We included 377 participants (193 control, 184 intervention) in the LCA with weight data available for 331 participants (87.8%; 167 control, 164 intervention) at 12 months, 283 participants (75.1%; 130 control 153 intervention) at 24 months, and 228 participants (60.5%; 122 control, 106 intervention) at 36 months (Fig. 1).

### Latent classes of infant dietary patterns at 10 months

A four-class solution was determined to be the best fitting model after comparing the fit indices of the latent class profile solutions (**Supplementary Table 1**) and inspecting latent class interpretability. The model fit indices supported the superiority of both the three-class and four-class over the two-class model, as evidenced by lower AIC, BIC, and ABIC values. Both the three-class and four-class models demonstrated a significant BLRT, indicating better fit compared to models with one fewer class. Although the four-class model did not have the lowest BIC, it was selected as the best-fitting model over the three-class model because all four classes contained more than 10% of the sample, had significant BLRT results, and exhibited the lowest AIC and ABIC values. Additionally, the four-class model allowed for the identification of distinct classes that captured the variety of complementary foods consumed. A five-class model was also examined but ultimately rejected due to an insignificant BLRT and the presence of a class comprising < 5% of the sample.

**Figure 2:**
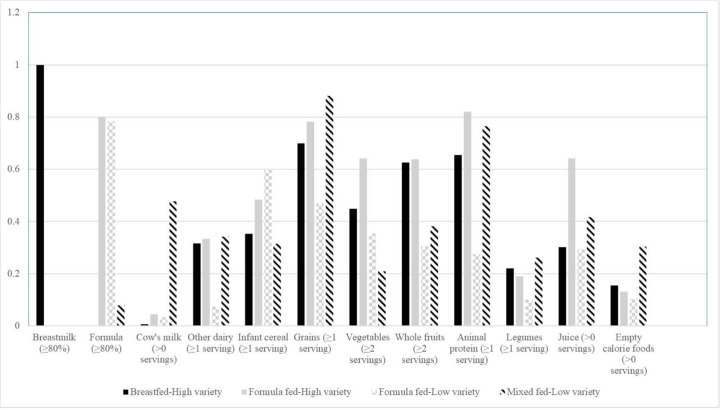
Daily food group item response probabilities for the four latent classes of infant dietary patterns at 10-months in the Starting Early Program Trial (N=377). Food group items that have a probability ≥0.5 characterize the dietary pattern.

Figure 2 shows the food group item response probabilities for each of the four latent classes. Food group item response probabilities closer to one indicated that the infant was more likely to consume that item, whereas probabilities closer to zero suggested that the infant was less likely to consume that item. Latent classes were labeled based on the high probability (≥ 0.50) of milk feeding mode and variety and developmental appropriateness of nutrient-dense complimentary foods (fruits, vegetables, grains, other dairy, animal protein, and legumes) served. The first class, labeled Breastfed-High variety (36.1% of the sample), was characterized by being predominantly breastfed and consuming a high variety of nutrient-dense complementary foods (fruits, grains, animal proteins). The second class, Formula fed-High variety (25.5%), had a high probability of being predominantly formula fed, consuming a high variety of complementary foods (grains, vegetables, fruits, animal protein), and having juice. The third class, Formula fed-Low Variety (24.1%), had a high probability of being predominantly formula fed and consuming infant cereal. The fourth class, Mixed fed-Low Variety (14.3%), had low probabilities of being predominantly breastfed or formula fed, suggesting mixed feeding, and a low variety of complementary foods (grains and animal proteins).

### Maternal and infant characteristics

**Table 1** shows maternal and infant characteristics. The Breastfed-High variety pattern had a greater proportion of mothers who were legally/living as married, born outside the U.S., and in the StEP intervention arm and a lower proportion of mothers who were employed or used childcare at 10 months. The Formula-fed High variety and Formula-fed low variety patterns had a greater proportion of mothers with a high school education or greater.

### Dietary patterns at 10 months and child weight outcomes at 12, 24, and 36 months

In unadjusted analyses, the Formula fed-High Variety and Formula fed-Low variety patterns were associated with greater WFAz at 24 and 36 months (**Supplementary Table 2**), but there were no associations with likelihood of being classified as overweight. In adjusted analyses (**Table 2**), compared to the Breastfed-High variety pattern, infants in the Formula fed-High variety pattern had higher WFAz at 24 months, and infants in the Formula fed-Low variety pattern had higher WFAz at 24 and 36 months and higher likelihood of being classified as overweight at 24 and 36 months. There was no association between the Mixed fed-Low variety pattern and weight status at 12, 24, or 36 months.

#### StEP intervention impacts on child weight outcomes at 12 and 24 months as mediated by infant dietary patterns

When testing the relationship between intervention group and dietary patterns, StEP participants were 2.4 (95% CI: 1.4, 4.1; *p* = 0.002) times more likely to be in the Breastfed-High variety pattern compared to the Formula Fed-High variety pattern. We found similar trends when comparing membership of StEP participants in the Breastfed-High variety pattern to the Formula Fed-Low variety pattern (OR: 1.5, 95% CI: 0.9, 2.6, *p* = 0.121) and the Mixed Fed-Low variety pattern (OR: 1.8, 95% CI: 0.9, 3.4, *p* = 0.074). Since there were no associations between intervention group and membership in any of the other dietary patterns, we dichotomized the dietary pattern variable for the mediation analysis as membership in the Breastfed-High variety pattern (yes or no). After transforming the variable, we found that StEP participants were 1.9 (95% CI: 1.2, 2.9; p = 0.004) times more likely to be in the Breastfed-High variety pattern compared to not being in this pattern.

**Figure 3:**
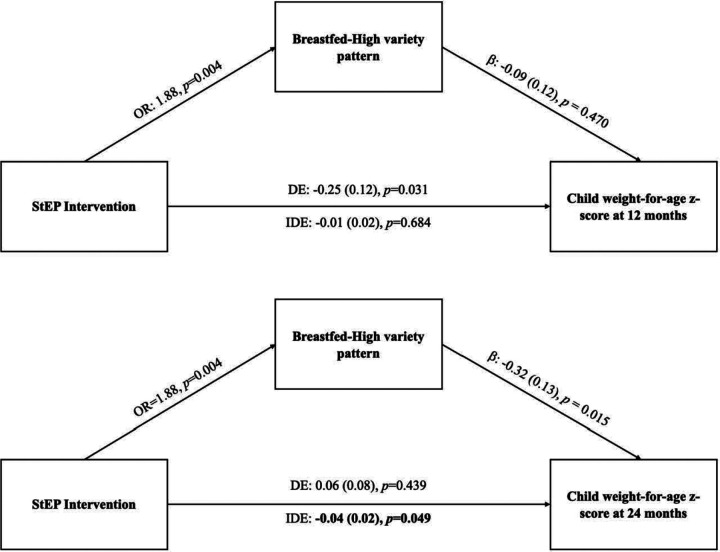
Indirect effects of the Starting Early Program Trial on child weight-for-age z-score at 12 and 24 months through dietary patterns at 10 months. StEP: Starting Early Program; OR: odds ratio; DE: direct effect; IDE: indirect effect. Mediation analysis at 24 months adjusted for weight-for-age z-score at 12 months

Figure 3 displays the effect of the StEP intervention on 10-month dietary patterns, the effect of membership in the Breastfed-High variety pattern on WFAz at 12 and 24 months, and the mediated effects of the StEP intervention on WFAz. Mediation analyses indicated a significant indirect pathway between the StEP intervention and WFAz at 24 months (Indirect Effect: −0.04, *p* = 0.049) via intervention impacts on infant dietary patterns. There was no indirect effect observed for WFAz at 12 months.

## Discussion

In this secondary analysis, we identified four infant dietary patterns: 1) Breastfed-High variety, 2) Formula fed-High variety, 3) Formula fed-Low variety, and 4) Mixed fed-Low variety. The Breastfed-High variety pattern was considered the most consistent with infant feeding guidelines because caregivers served a high variety of nutrient-dense complementary foods and intakes of juice and empty calorie foods were low. Although this pattern was not characterized by ≥ 2 servings of vegetables, most infants received ≥ 1 serving, and vegetable intakes were higher than the Formula fed-Low variety and Mixed fed-Low variety patterns. Compared to the Breastfed-High variety pattern, infants in the Formula fed-High variety pattern had higher WFAz at 24 months, and infants in the Formula fed-Low variety pattern had higher WFAz and greater likelihood of being classified as overweight at 24 and 36 months. StEP participants were more likely to be in the Breastfed-High variety pattern, and participation in StEP was significantly indirectly associated with lower WFAz at 24 months through greater membership in this dietary pattern.

Compared to data from national surveys, infants in the current analysis had similar or higher rates of breastfeeding and fruit intake; similar or lower rates of formula feeding and juice consumption; similar rates of vegetable intake; and lower consumption of empty calorie foods (12, 18). However, average dietary intakes did not meet recommendations. Most infants were not receiving recommended amounts of fruits, vegetables, and other dairy based on CACFP guidelines (27), just under half of infants were given juice, and consumption of cow’s milk and empty calorie foods was evident, which may have implications for future dietary habits, food preferences, and health.

A main difference between the infant dietary patterns identified in our study compared to other research was the absence of a pattern characterized by empty calorie foods (33). While the reason for this difference is unclear, participants in the current analysis self-identified as Hispanic or Latina, and most were born outside of the U.S. In qualitative studies, Hispanic or Latina mothers born outside the U.S. reported balancing cultural- and family-based feeding recommendations with evidence-based health information when making decisions about infant feeding, with unprocessed, natural, and homemade cultural foods perceived as the healthiest options (34–36). Additionally, most of the families in our study participated in WIC, which aligns to national dietary guidelines and is associated with higher infant and child diet quality (37).

Prior research found that infant dietary patterns characterized by empty calorie foods, juice, and/or low variety of complementary foods were associated with increased child weight outcomes at 2 and 6 years (19, 20). While our study found that dietary patterns characterized by juice and low variety were associated with increased weight, we interestingly did not find any associations with the Mixed fed-Low variety pattern. While nearly one-third of infants in this pattern had ≥ 1 serving of empty calorie foods, this proportion is lower than what is reported in research that found associations with weight (12, 18–20). Additionally, most infants in the Mixed fed-Low variety pattern received some breastmilk, which may have a protective effect on weight (38, 39). The exact reasoning for the lack of association is unclear; unmeasured confounding associated with breastfeeding may have occurred. Infant dietary patterns that are characterized by a low variety of nutrient-dense complementary foods limit exposure to flavors and textures, which may prevent food acceptance later in childhood and lead to unhealthy food preferences (40–42). Continued exposure to these dietary patterns increases risks of nutritional deficiencies and obesogenic eating behaviors (43–45), potentially promoting excess energy intake, weight gain, and adiposity (33, 46).

Of the dietary patterns identified, the Breastfed-High variety pattern was considered the most consistent with infant feeding guidelines. StEP participants were more likely to follow the Breastfed-High variety dietary pattern, suggesting that StEP fostered adoption of guideline-concordant feeding behaviors. This finding aligns with the StEP curriculum, which promotes breastfeeding, optimal infant feeding practices, and modeling healthy behaviors, as well as prior research on the impacts of StEP on infant feeding behaviors, including greater maternal feeding knowledge and style, increased breastfeeding, and decreased juice (22). We also documented indirect effects of StEP on WFAz at 24 months, but not 12 months, through intervention impacts on membership in the Breastfed-High variety pattern. As milk-based feeding is still a significant form of energy in infants through 12 months, we did not expect that dietary patterns at 10 months would be strongly associated with 12-month WFAz. Rather, we conceptualized that infant dietary patterns may influence later child weight by establishing dietary patterns that continue throughout childhood (47, 48). StEP may have facilitated continued support to maintain guideline-concordant dietary patterns beyond infancy, but additional research is needed.

Strengths of this analysis include LCA to assess the combination of infant feeding and type and variety of complementary foods in Hispanic and low-income families. Limitations include using a single 24-hour recall to assess infant dietary intake, which may not account for day-to-day variation or reflect usual intake (49). This limitation may be partly alleviated because mothers were asked to report what their infant ate on a typical day. LCA did not take into account early infant feeding practices, such as breastfeeding and formula feeding duration, intensity, or exclusivity for the first 6 months, time of introduction of complementary foods, or variety of complementary foods beyond what was reported on a single day. Additionally, our study included a culturally diverse sample of Hispanic and low-income families who were from an urban setting, which may not be generalizable to other populations.

In conclusion, this study suggests that dietary patterns that are inconsistent with infant feeding guidelines, particularly low variety of nutrient-dense complementary foods, are associated with increased early childhood weight outcomes. StEP promoted a dietary pattern that was most consistent with infant feeding guidelines, which mediated the intervention effects on WFAz at 24 months. Given the importance of early prevention, culturally-relevant interventions that promote healthy nutrition and lifestyle behaviors in infancy may encourage families to provide a variety of developmentally appropriate and nutrient-dense complementary foods that promote health. More research is warranted to understand how dietary patterns change throughout childhood in Hispanic and low-income populations, determine factors that influence dietary patterns, and examine associations with weight outcomes.

## Supplementary Material

Supplementary Files

This is a list of supplementary files associated with this preprint. Click to download.

- IJOSupplementalTable111.07.25.docx
- IJOTable1.docx
- IJOSupplementalTable211.07.25.docx
- IJOTable2.docx

Tables 1 and 2 are available in the Supplementary Files section.

## Data Availability

The datasets generated during and/or analyzed during the current study are available from the corresponding author on reasonable request.

## Abbreviations

U.S.: United States
LCA: latent class analysis
StEP: Starting Early Program
WFAz: weight-for-age z-score
ASA24: Automated Self-Administered 24-Hour Dietary Assessment 2014
NCI: National Cancer Institute
FPED: Food Pattern Equivalents Database
CACFP: Child and Adult Care Food Program
EMR: electronic medical record
WIC: Special Supplemental Nutrition Program for Women, Infants, and Children
BMI: body mass index
AIC: Akaike’s Information Criterion
BIC: Bayesian Information Criterion
ABIC: adjusted Bayesian Information Criterion
BLRT: parametric bootstrapped likelihood ratio test

## References

[1] Stierman B, Afful J, Carroll MD, Chen T-C, Davy O, Fink S, National Health and Nutrition Examination Survey 2017–March 2020 prepandemic data files development of files and prevalence estimates for selected health outcomes. Natl Health Stat Report. 2021; 14(158):10.15620/cdc:106273. PMC11513744 39380201

[2] Fryar C, Carroll M, Afful J. Prevalence of overweight, obesity, and severe obesity among adults aged 20 and over: United States, 1960–1962 through 2017–2018. NCHS Health E-Stats. 2020. https://www.cdc.gov/nchs/data/hestat/obesity-adult-17-18/obesity-adult.htm#Citation. Accessed November 21, 2022.

[3] Simmonds M, Llewellyn A, Owen CG, Woolacott N. Predicting adult obesity from childhood obesity: a systematic review and meta-analysis. Obes Rev. 2016;17(2):95–107. doi:10.1111/obr.12334. 26696565

[4] Bianco-Miotto T, Craig JM, Gasser YP, van Dijk SJ, Ozanne SE. Epigenetics and DOHaD: from basics to birth and beyond. J Dev Orig Health Dis. 2017;8(5):513–9. doi:10.1017/s2040174417000733. 28889823

[5] Woo Baidal JA, Locks LM, Cheng ER, Blake-Lamb TL, Perkins ME, Taveras EM. Risk factors for childhood obesity in the first 1,000 days: a systematic review. Am J Prev Med. 2016;50(6):761–79. doi:10.1016/j.amepre.2015.11.012. 26916261

[6] Hamner HC, Nelson JM, Sharma AJ, Jefferds MED, Dooyema C, Flores-Ayala R, Improving Nutrition in the First 1000 Days in the United States: A Federal Perspective. Am J Public Health. 2022;112(S8):S817–s25. doi:10.2105/ajph.2022.307028. 36122314 PMC9612192

[7] Ong KK. Healthy Growth and Development. Nestle Nutr Inst Workshop Ser. 2017;87:141–51. doi:10.1159/000448964. 28315895

[8] Birch LL, Doub AE. Learning to eat: birth to age 2 y. Am J Clin Nutr. 2014;99(3):723S–8S. doi:10.3945/ajcn.113.069047. 24452235

[9] U.S Department of Agriculture and U.S. Department of Health and Human Services. Dietary Guidelines for Americans, 2020–2025. 9th Edition. December 2020. https://www.dietaryguidelines.gov/sites/default/files/2020-12/Dietary_Guidelines_for_Americans_2020-2025.pdf. Accessed January 18, 2023.

[10] Breastfeeding and the use of human milk. Pediatrics. 2012; 129(3):e827–41. 10.1542/peds.2011-3552. 22371471

[11] Bailey RL, Stang JS, Davis TA, Naimi TS, Schneeman BO, Dewey KG, Dietary and complementary feeding practices of US infants, 6 to 12 months: a narrative review of the federal nutrition monitoring data. J Acad Nutr Diet. 2022;122(12):2337–45.e1. doi:10.1016/j.jand.2021.10.017. 34688966 PMC10851078

[12] Roess AA, Jacquier EF, Catellier DJ, Carvalho R, Lutes AC, Anater AS, Food Consumption Patterns of Infants and Toddlers: Findings from the Feeding Infants and Toddlers Study (FITS) 2016. J Nutr. 2018;148(suppl_3):1525s–35s. doi:10.1093/jn/nxy171. 30247583 PMC6126630

[13] Qiao J, Dai LJ, Zhang Q, Ouyang YQ. A meta-analysis of the association between breastfeeding and early childhood obesity. J Pediatr Nurs. 2020;53:57–66. doi:10.1016/j.pedn.2020.04.024. 32464422

[14] Dewey K, Bazzano L, Davis T, Donovan S, Taveras E, Kleinman R, The duration, frequency, and volume of exclusive human milk and/or infant formula consumption and overweight and obesity: a systematic review. Alexandria (VA): USDA Nutrition Evidence Systematic Review; 2020. 35315996

[15] English LK, Obbagy JE, Wong YP, Butte NF, Dewey KG, Fox MK, Types and amounts of complementary foods and beverages consumed and growth, size, and body composition: a systematic review. Am J Clin Nutr. 2019;109(Suppl_7):956s–77s. doi:10.1093/ajcn/nqy267. 30982866

[16] English LK, Obbagy JE, Wong YP, Butte NF, Dewey KG, Fox MK, Timing of introduction of complementary foods and beverages and growth, size, and body composition: a systematic review. Am J Clin Nutr. 2019;109(Suppl_7):935s–55s. doi:10.1093/ajcn/nqy267. 30982863

[17] Reedy J, Subar AF, George SM, Krebs-Smith SM. Extending methods in dietary patterns research. Nutrients. 2018;10(5). doi:10.3390/nu10050571. PMC5986451 29735885

[18] Rose CM, Savage JS, Birch LL. Patterns of early dietary exposures have implications for maternal and child weight outcomes. Obesity (Silver Spring, Md). 2016;24(2):430–8. 26717908 10.1002/oby.21349

[19] Rose CM, Birch LL, Savage JS. Dietary patterns in infancy are associated with child diet and weight outcomes at 6 years. International journal of obesity (2005). 2017;41(5):783–8. doi:10.1002/oby.21349. 28133360

[20] Hohman EE, Paul IM, Birch LL, Savage JS. INSIGHT responsive parenting intervention is associated with healthier patterns of dietary exposures in infants. Obesity (Silver Spring, Md). 2017;25(1):185–91. doi:10.1038/ijo.2017.27. 28008749 PMC5189916

[21] Messito MJ, Mendelsohn AL, Katzow MW, Scott MA, Vandyousefi S, Gross RS. Prenatal and pediatric primary care-based child obesity prevention program: a randomized trial. Pediatrics. 2020;146(4). doi:10.1542/peds.2020-0709. PMC7546096 32883807

[22] Messito MJ, Katzow MW, Mendelsohn AL, Gross RS. Starting Early Program impacts on feeding at infant 10 months age: a randomized controlled trial. Child Obes. 2020;16(S1):S4–s13. doi:10.1089/chi.2019.0236. 31934788 PMC7469695

[23] Gross RS, Mendelsohn AL, Gross MB, Scheinmann R, Messito MJ. Randomized controlled trial of a primary care-based child obesity prevention intervention on infant feeding practices. J Pediatr. 2016;174:171–7.e2. doi:10.1016/j.jpeds.2016.03.060. 27113376 PMC4925185

[24] Kim CN, Messito MJ, Katzow M, Duh-Leong C, Gross RS. Child obesity prevention from pregnancy: long-term follow-up of the Starting Early Program Trial. Pediatrics. 2025;155(5). doi:10.1542/peds.2024-069421. PMC13007116 40164193

[25] Subar AF, Kirkpatrick SI, Mittl B, Zimmerman TP, Thompson FE, Bingley C, The Automated Self-Administered 24-hour dietary recall (ASA24): a resource for researchers, clinicians, and educators from the National Cancer Institute. J Acad Nutri Diet. 2012;112(8):1134–7. doi:10.1016/j.jand.2012.04.016. PMC3721511 22704899

[26] National Cancer Institute. Calculating Food Patterns Equivalents Database (FPED) Food Group Equivalents. Reviewing and Cleaning ASA24 Data. https://epi.grants.cancer.gov/asa24/resources/cleaning.html#calculate. Accessed February 21, 2019.

[27] United States Department of Agriculture. Child and Adult Care Food Program (CACFP) Infant Meal Patterns. 2022. https://www.fns.usda.gov/cacfp/nutrition-standards/infant-meal-patterns. Accessed February 21, 2019.

[28] Mennella JA, Ziegler P, Briefel R, Novak T. Feeding Infants and Toddlers Study: the types of foods fed to Hispanic infants and toddlers. J Am Diet Assoc. 2006;106(1 Suppl 1):S96–106. doi:10.1016/j.jada.2005.09.038. 16376634

[29] Li R, Fein SB, Grummer-Strawn LM. Association of breastfeeding intensity and bottle-emptying behaviors at early infancy with infants’ risk for excess weight at late infancy. Pediatrics. 2008;122 Suppl 2:S77–84. doi:10.1542/peds.2008-1315j. 18829835

[30] World Health Organization. World Health Organization anthro for personal computers, version 3.2.2, 2011: software for assessing growth and development of the world’s children. 2011. http://www.who.int/childgrowth/software/en/. Accessed August 21, 2018.

[31] Bickel G, Nord M, Price C, Hamilton W, Cook J. Guide to Measuring Household Food Security (Revised 2000). USDA Food and Nutrition Service. 2020. https://www.fns.usda.gov/research/guide-measuring-household-food-security-revised-2000. Accessed August 21, 2018.

[32] Nylund KL, Tihomir A, and Muthén BO. Deciding on the number of classes in latent class analysis and growth mixture modeling: a Monte Carlo simulation study. Struct Equ Modeling. 2007;14(4):535–69. doi:10.1080/10705510701575396.

[33] Thompson AL. Evaluating the pathways linking complementary feeding practices to obesity in early life. Nutr Rev. 2020;78(Suppl 2):13–24. doi:10.1093/nutrit/nuz057. PMC9633281 33196090

[34] MacMillan Uribe AL, Rudt HG, Leak TM. Cultural influences on infant and toddler feeding among low-income Latinx mothers. Matern Child Nutr. 2022;18(4):e13342. doi:10.1111/mcn.13342. 35702987 PMC9480920

[35] Beck AL, Hoeft KS, Takayama JI, Barker JC. Beliefs and practices regarding solid food introduction among Latino parents in Northern California. Appetite. 2018;120:381–7. doi:10.1016/j.appet.2017.09.023. 28951238 PMC5784836

[36] Cheney AM, Nieri T, Davis E, Prologo J, Valencia E, Anderson AT, The sociocultural factors underlying Latina mothers’ infant feeding practices. Glob Qual Nurs Res. 2019;6:2333393618825253. doi:10.1177/2333393618825253. 30746425 PMC6360473

[37] Caulfield LE, Bennett WL, Gross SM, Hurley KM, Ogunwole SM, Venkataramani M, Maternal and child outcomes associated with the Special Supplemental Nutrition Program for Women, Infants, and Children (WIC). Agency for Healthcare Research and Quality Comparative Effectiveness Reviews. Rockville (MD): Agency for Healthcare Research and Quality (US); 2022. 35503870

[38] Koletzko B, Godfrey KM, Poston L, Szajewska H, van Goudoever JB, de Waard M, Nutrition during pregnancy, lactation and early childhood and its implications for maternal and long-term child health: the Early Nutrition project recommendations. Ann Nutr Metab. 2019;74(2):93–106. doi:10.1159/000496471. 30673669 PMC6397768

[39] Ventura AK. Does breastfeeding shape food preferences? links to obesity. Ann Nutr Metab. 2017;70 Suppl 3:8–15. doi:10.1159/000478757. 28903109

[40] Anzman-Frasca S, Ventura AK, Ehrenberg S, Myers KP. Promoting healthy food preferences from the start: a narrative review of food preference learning from the prenatal period through early childhood. Obes Rev. 2018;19(4):576–604. doi:10.1111/obr.12658. 29266778

[41] Maier-Nöth A. Early development of food preferences and healthy eating habits in infants and young children. Nestle Nutr Inst Workshop Ser. 2019;91:11–20. doi:10.1159/000493674. 30865954

[42] Emmett PM, Hays NP, Taylor CM. Antecedents of picky eating behaviour in young children. Appetite. 2018;130:163–73. doi:10.1016/j.appet.2018.07.032. 30099068 PMC6173797

[43] Evans C, Hutchinson J, Christian MS, Hancock N, Cade JE. Measures of low food variety and poor dietary quality in a cross-sectional study of London school children. Eur J Clin Nutr. 2018;72(11):1497–505. doi:10.1038/s41430-017-0070-1. 29391590

[44] Steyn NP, Nel JH, Nantel G, Kennedy G, Labadarios D. Food variety and dietary diversity scores in children: are they good indicators of dietary adequacy? Public Health Nutr. 2006;9(5):644–50. 16923296 10.1079/phn2005912

[45] Taylor CM, Wernimont SM, Northstone K, Emmett PM. Picky/fussy eating in children: review of definitions, assessment, prevalence and dietary intakes. Appetite. 2015;95:349–59. doi:10.1016/j.appet.2015.07.026. 26232139

[46] Thompson AL, Bentley ME. The critical period of infant feeding for the development of early disparities in obesity. Soc Sci Med. 2013;97:288–96. doi:10.1016/j.socscimed.2012.12.007. 23312304 PMC3812266

[47] Lioret S, Betoko A, Forhan A, Charles MA, Heude B, de Lauzon-Guillain B. Dietary patterns track from infancy to preschool age: cross-sectional and longitudinal perspectives. J Nutr. 2015;145(4):775–82. doi:10.3945/jn.114.201988. 25833780

[48] Luque V, Escribano J, Closa-Monasterolo R, Zaragoza-Jordana M, Ferré N, Grote V, Unhealthy dietary patterns established in infancy track to mid-childhood: the EU Childhood Obesity Project. J Nutr. 2018;148(5):752–9. doi:10.1093/jn/nxy025. 29982656

[49] Thompson FE, Kirkpatrick SI, Subar AF, Reedy J, Schap TE, Wilson MM, The National Cancer Institute’s Dietary Assessment Primer: a resource for diet research. J Acad Nutr Diet. 2015;115(12):1986–95. doi:10.1016/j.jand.2015.08.016. 26422452 PMC4663113

